# Trauma-induced coagulopathy and critical bleeding: the role of plasma and platelet transfusion

**DOI:** 10.1186/s40560-016-0203-y

**Published:** 2017-01-20

**Authors:** Hiroyasu Ishikura, Taisuke Kitamura

**Affiliations:** Department of Emergency and Critical Care Medicine, Faculty of Medicine, Fukuoka University, 7-45-1 Nanakuma, Jonan-ku, Fukuoka, 814-0180 Japan

**Keywords:** Transfusion, Plasma, Fresh frozen plasma, FFP, Platelet, Trauma, Damage control resuscitation, Acute traumatic coagulopathy, Acute coagulopathy of trauma shock, Trauma-induced coagulopathy

## Abstract

Hemorrhage is responsible for 30 to 40% of all trauma-related mortality. Among adult trauma patients, 94% of hemorrhage-related deaths occur within 24 h and approximately 60% of these deaths within 3 h of hospital admission. Therefore, appropriate initial fluid resuscitation for bleeding is crucial to avoid preventable trauma-related death. In particular, the resuscitation strategy must be designed to complement prompt correction of anemia, coagulopathies, and thrombocytopenia. Conventional damage control resuscitation (DCR) of patients with severe trauma and massive hemorrhage is usually begun with rapid infusion of 1000 to 2000 mL of crystalloid fluids with subsequent transfusion of type O or uncross-matched red blood cells (RBCs) without plasma such as fresh frozen plasma (FFP) or platelets (PLTs). However, this DCR technique often leads to several adverse events such as abdominal compartment syndrome, acute respiratory distress syndrome, multiple organ failure, and dilutional coagulopathy. Simultaneous transfusion of FFP and PLTs along with the first units of RBCs while minimizing crystalloid infusion was recently recommended as a renewed DCR strategy. This aggressive RBC transfusion with FFP and PLTs is not only essential for the correction of coagulopathies and thrombocytopenia but also has the potential to ensure a good outcome in trauma patients. Additionally, it is important to maintain the resuscitation ratios of FFP/RBC and PLT/RBC. Most recently, DCR has been advocated for rapid hemorrhage control through early administration of a mixture of FFP, PLTs, and RBCs in a balanced ratio of 1:1:1.

## Background

Trauma is a major health care issue that results in the annual death of 5–8 million people worldwide [1]. It is the sixth most common cause of death in Japan and the third most common in the USA. Hemorrhage is responsible for 30–40% of total trauma-related mortality [2]. Among adult trauma patients, 94% of hemorrhagic deaths occur within 24 h and ~60% of these deaths within 3 h of hospital admission [3].

Bleeding in trauma is due to vascular damage, but in 25–30% of patients, it is also due to trauma-induced coagulopathy [1, 4–6]. It was long presumed that the main causative factor in traumatic coagulopathy was iatrogenic hemodilution. However, traumatic coagulopathy was recently shown to already develop at the trauma scene, before any medical intervention [7]. This finding is of particular relevance given that traumatic coagulopathy results in considerably increased mortality [4, 5, 8, 9].

In the early phase of injury, rapid surgical or angiographic hemostasis is the first priority in avoiding trauma-related death. Preventable trauma death (PTD) may be attributable to the absence of appropriate initial resuscitation for bleeding. Therefore, any effective resuscitation strategy must be designed to complement the appropriate and prompt correction of anemia, coagulopathy, and abnormalities in fibrinolysis.

The damage control resuscitation (DCR) strategy, which is focused on halting and/or preventing the lethal triad of coagulopathy, acidosis, and hypothermia, has challenged traditional thinking on early resuscitation strategies [10]. In DCR, transfusion is carried out during the early stage of patient management. It involves the use of increased amounts of plasma and platelets (PLTs) along with the first units of red blood cells (RBCs), while simultaneously minimizing crystalloid administration in patients who are predicted to require massive transfusion (defined as >10 units of RBCs in 24 h) [10–13].

## Coagulopathy during the pre-hospital stage

Our understanding of major hemorrhage in trauma has dramatically changed over the past decade, mainly due to the recognition that patients who are bleeding when they present to the hospital already have an established coagulopathy, before the dilutional effects of fluid resuscitation. This has led to the use of a new terminology: acute traumatic coagulopathy (ATC) or acute coagulopathy of trauma shock, or trauma-induced coagulopathy (TIC). ATC/TIC quickly follows severe and profound injury and is present in one quarter to one third of these patients at the time of hospital admission [14, 15].

The importance of ATC/TIC is that its presence is a prognostic indicator, based on its association with a poor clinical outcome. In traumatic hemorrhage patients, a prolonged prothrombin time (PT) and/or activated partial thromboplastin time (aPTT) at hospital admission, before resuscitation, is associated with a three- to fourfold higher mortality and independently associated with increased transfusion requirements, organ dysfunction, and critical care length of stay [16, 17].

ATC/TIC is a multifactorial, global failure of the coagulation system to sustain adequate hemostasis after major trauma. Its pathophysiology is thought to be due to the massive stimulation of thrombin generation, PLT consumption, and fibrinolysis by damaged tissues [18, 19]. Tissue damage exposes tissue factor, which drives thrombin generation and activation of the coagulation cascade. Thrombin activates PLTs, leukocytes, tissue plasminogen activator (t-PA), and the endothelium. Other factors that activate t-PA include hypoxia and vasopressors [17].

## The development of coagulopathy in the emergency department

In the 1970s and 1980s, resuscitation of the most severely injured and massively hemorrhaging patients usually began with the rapid administration of 1000–2000 mL of crystalloid fluids, followed by type O or uncross-matched RBCs. However, the administration of significant amounts of crystalloid leads to abdominal compartment syndrome, acute respiratory distress syndrome (ARDS), and multiple organ failure [20]. Ley et al. [21] determined that the replacement of ≥1.5 L of intravenous crystalloids in the emergency department is an independent risk factor for mortality. High volumes of crystalloids (>3 L) are associated with a high mortality rate, particularly in elderly trauma patients. Additionally, in this setting, low blood volume, insensible losses and/or consumption, and resuscitation with plasma-poor RBCs rapidly lead to plasma coagulation factor concentrations of <40%, even before 10 units of RBCs have been transfused. However, the early initiation of plasma therapy is often delayed by its lack of immediate availability in the trauma center. Additionally, while PLT concentrations usually fall to 50–100 × 10^9^/L (=50,000–100,000/μL) after 10–20 units of RBCs have been administered, in individual patients, they are quite variable and larger decreases are possible [22]. Consequently, delays in the early initiation of PLT therapy are typically greater than those in plasma administration.

## Situation of dilutional coagulopathy and thrombocytopenia according to differences in blood components

**Fig. 1:**
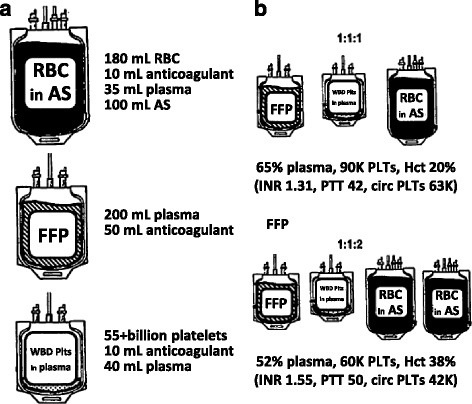
Conventional blood products and effects of administering them in ratios. **a** Composition of standard units of the following blood components: RBCs, FFP, and WBD PLTs. All PLT units in the Pragmatic Randomized Optimal Plasma and Platelet Ratios (PROPPR) study and 85% of PLTs used in the USA are in the form of apheresis units equal to six WBD units. The 55 billion PLTs in one WBD PLT unit occupy <0.5 mL. **b** Composition of the constituents in 1:1:1 and 1:1:2 mixtures of FFP, WBD PLTs, and RBC units. The *top row* is calculated directly from the contents, reflecting the extent to which anticoagulant and RBC additive solution dilute plasma and PLTs and RBCs are diluted by mixing with the other components. In the *bottom row*, the international normalized ratio and partial thromboplastin time values come from the experimental data of Kornblith et al. [23], whereas the circulating PLT counts given are 70% of the infused PLT counts to reflect the poor recovery of stored PLTs. Reproduced with permission [12]. *RBCs* red blood cells, *FFP* fresh frozen plasma, *WBD* whole blood-derived, *PLTs* platelets

Kornblith et al. [23] obtained 23 units of fresh frozen plasma (FFP), PLT concentrate, and RBCs from a regional blood collection center and mixed them to create 23 units of 1:1:1 and 1:1:2 reconstituted whole blood (RWB) (Fig. 1). They then measured the international normalized ratio (INR)/partial thromboplastin time (PTT) and performed a complete blood cell count, functional studies, and an extensive panel of procoagulant and anticoagulant factor assays using these products. The hemoglobin (Hgb) and hematocrit (Hct) were significantly lower in patients administered 1:1:1 RWB than 1:1:2 RWB, but the former had a higher PLT count. Moreover, the PLT counts were typically only 70% of the transfused circulating PLTs. Notably, 1:1:1 RWB patients had a significantly lower INR and PTT than did 1:1:2 RWB patients and a significantly higher fibrinogen level (Table 1). Previous studies [24, 25] showed that an INR or PTT ratio >1.5 and low PLT counts were strongly associated with uncontrolled microvascular bleeding and hemorrhage-related mortality [26]. Therefore, caution is warranted regarding the use of therapy with blood components, as an inappropriate unit ratio will lead to dilutional coagulopathy and thrombocytopenia.

**Table 1:** Clotting profiles by RWB variants (1:1:1 vs. 2:1:1)

	1:1:1		2:1:1		
	*n* = 23	SD	*n* = 23	SD	*p*
INR	1.31	0.18	1.55	0.31	0.0029
PT, s	15.98	1.71	18.20	2.75	0.0024
PTT, s	41.76	4.68	49.92	8.12	0.0008
Hgb, g/dL	9.05	1.07	12.15	1.22	0.0000
Hct, %	28.93	3.46	39.19	3.90	0.0000
PLTs, ×10^9^/L	129.62	22.23	95.48	21.85	0.0000

Data are given as mean and SD. Significance assessed by Student’s *t* test for normally distributed data and Wilcoxon rank-sum test for nonnormally distributed data

*RWB* reconstituted whole blood, *INR* international normalized ratio, *PT* prothrombin time, *PTT* partial thromboplastin time, *Hgb* hemoglobin, *Hct* hematocrit, *PLTs* platelets

*p* < 0.05, significant

## FFP transfusion

### Colloid or crystalloid in DCR?

The overuse of crystalloids before the administration of any blood product as a primary initial resuscitative solution in patients with hemorrhagic shock leads to dilutional coagulopathy, pulmonary edema (ARDS), and severe interstitial edema (abdominal compartment syndrome). Furthermore, because crystalloid- or colloid-based resuscitation causes acidosis, and a steady decline in oxygen delivery, the underlying coagulation and metabolic disorders that evolve after injury and blood loss are further aggravated [27].

However, for the last four decades, most trauma resuscitations have consisted of an initial 2000 mL of crystalloid given according to the early protocols of the American College of Surgeons’ Advanced Trauma Life Support course. During the same period, there was little argument about PLT administration. However, this resulted in unintentional hemodilution, which led to a vicious circle of coagulopathy, acidosis, and hypothermia, the “lethal triad of trauma” [28].

### Importance of FFP transfusion and the FFP/RBC ratio

Cinat et al. [29] reported that early aggressive transfusion with FFP is essential to the correction of coagulopathy and results in a good outcome in trauma patients. According to Hirshberg et al. [30], prolongation of the PT to >1.8 times normal is the sentinel event of dilutional coagulopathy and the key to preventing coagulopathy is plasma infusion before the PT becomes subhemostatic. Based on a computer simulation, those authors concluded that the optimal replacement FFP/RBC and PLT/RBC ratios were 2:3 for plasma and 8:10 for PLTs to minimize dilutional coagulopathy. In the early 2000s, the two-part DCR concept was proposed for severely injured patients. The strategy is initiated within minutes of the arrival of these patients in the emergency department, with resuscitation initially limited to maintaining blood pressure at ~90 mmHg; this so-called permissive hypotension prevents renewed bleeding from recently clotted vessels. In the second step, intravascular volume restoration is accomplished using plasma as the primary resuscitation fluid in at least a 1:1 or 1:2 ratio of FFP/RBC [13].

Many studies have evaluated the effects of the FFP/RBC on mortality in massive transfusion after trauma (Table 2). Most of them demonstrated a survival advantage of an increased plasma ratio, with the majority suggesting an optimal FFP/RBC ratio of ≥1:2.

**Table 2:** Effects of FFP/RBC ratio on mortality outcome

Authors	Year	Patients	FFP/RBC ratio	Results
Borgman	2007	246	<1:4 vs. 1:4—1:2 vs. >1:2	Improved in-hospital survival in highest ratio group (OR 8.6, CI 2.1–35.2)
Maegele	2008	713	<0.9 vs. 0.9—1.1 vs. >1.1	Decreased 30-day mortality in highest ratio group (24 vs. 46%, *p* < 0.001)
Duchesne	2008	135	<1:2 vs. >1:2	Increased mortality in low-ratio group (RR 18.9; CI 6.3–56.4)
Holcomb	2008	466	<1:2 vs. ≥1:2	Improved 30-day survival in high plasma transfusion group (73 vs. 43%, *p* < 0.001)
Zink	2009	466	<1:4 vs. 1:4—1:1 vs. ≥1:1	Decreased in-hospital mortality in high plasma transfusion groups (ARR of 29%)
Sperry	2008	415	<1:1.5 vs. ≥1:1.5	High plasma ratio is independently associated with lower mortality (HR 0.48, CI 0.3–0.8)
Gunter	2008	259	<2:3 vs. ≥2:3	Decreased 30-day morality in high plasma transfusion groups
Kashuk	2008	133	1:1 vs. 1:2 vs. 1:3 vs. 1:4 vs. ≥1:5	The lowest predicted mortality probability correlates with a plasma/red cell ratio of 1:2 and 1:3.
Teixeira	2009	383	≤1:8 vs. 1:8—1:3 vs. 1:3—1:2 vs. >1:2	Linear correlation between survival and plasma transfusion ratio. No survival advantage after plasma transfusion ratio reaches 1:3

However, this approach has not been tested in prospective randomized controlled trials.

The Prospective, Observational, Multicenter, Major Trauma Transfusion (PROMMTT) Study Group, a highly publicized prospective cohort study, documented the timing of transfusions during active resuscitation and the respective patient outcomes [3]. In the first 6 h after admission, patients with an FFP/RBC ratio of <1:2 were three to four times more likely to die than patients with ratios of ≥1:1. Higher plasma ratios early in resuscitation were associated with a decreased mortality in patients who received transfusions of at least three total units of RBCs, FFP, or PLTs during the first 24 h after admission. In a multivariable time-dependent Cox model, an increased FFP/RBC ratio was independently associated with a decrease in 6-h post-admission mortality (adjusted hazard ratio = 0.31; 95% confidence interval, 0.16–0.58), when hemorrhagic death predominates. Hagiwara et al. [31] also reported that in severe blunt trauma patients (injury severity score ≥16), the transfusion of an FFP/RBC ratio of ≥1 within the first 6  h was related to outcome. These results provide strong support for the early and high-dose administration of FFP in DRC blood transfusion therapy.

## Platelet transfusion

In severe trauma patients, massive transfusions of RBCs and FFP and other intravenous fluids give rise to dilutional thrombocytopenia. However, many studies have shown that in the early stages of trauma hemorrhage thrombocytes are maintained at levels not expected to contribute to a clinically significant coagulopathy [32]. Thus, while PLT transfusion may not be essential for the correction of ATC/TIC, together with the combined effects of shock, hypothermia, etc., ATC theoretically produces aberrant PLT function by disrupting activation and adhesion pathways.

### Starting time of platelet transfusion

In Japan, there are no clear criteria regarding the starting time of PLT transfusion for trauma patients, whereas in Europe, there is a guideline for the management of bleeding and coagulopathy following major trauma. According to the European guideline, in grade 1C patients, PLTs should be administered to maintain a concentration of >50 × 10^9^/L. In grade 2C patients, the maintenance of a PLT count of >100 × 10^9^/L is recommended for patients with ongoing bleeding and/or traumatic brain injury, with an initial dose of four to eight single PLTs units or one apheresis pack [1]. The guideline for the administration of PLTs, however, is based mainly on observational studies and opinions.

Ciavarella et al. [33] reported that the most sensitive laboratory predictors of diffuse non-mechanical microvascular bleeding (MVB) were a PLT count <50 × 10^9^/L (=50,000/μL) or a fibrinogen level of <0.5 g/L. Accordingly, a PLT count of <50 × 10^9^/L (=50,000/μL) may serve as one of the criteria for the starting time of PLT administration in severe trauma patients, even in the absence of MVB. This starting time is consistent with the study by Johansson et al. [34] of patients with a ruptured abdominal aortic aneurysm, in whom 30-day survival was related to the PLT count determined upon arrival at the intensive care unit (ICU); this PLT count in the analyzed patients was well above the recommended 50 × 10^9^/L.

However, some patients are able to maintain high PLT counts despite ongoing blood loss by recruiting PLTs from the spleen and possibly mobilizing new ones from bone marrow. Thus, when >220% of blood volume has been replaced, PLT counts will usually have fallen to around 50 × 10^9^/L and frank coagulopathy will ensue [35].

Based on their small prospective study of 27 patients requiring massive transfusion, Counts et al. [36] concluded that the most useful parameter for estimating the need for PLT transfusions was the PLT count, with a count as high as 100 × 10^9^/L potentially required to control bleeding from surgical wounds and serving as the threshold level for starting PLT transfusion.

A PLT count of <100 × 10^9^/L is a possible risk factor for mortality, and a PLT count of <50 × 10^9^/L leads to lethal coagulopathy. Therefore, PLT administration should be started when the PLT count is <100 × 10^9^/L, and it should be maintained at >50 × 10^9^/L in patients with severe trauma and/or ongoing bleeding. However, in one study, only 3% of trauma patients had a PLT count of <100 × 10^9^/L at ICU admission [37], so a delay in the start of platelet administration should be avoided.

### Ratio of the platelet transfusion

In massive transfusions (>10 units of RBCs within 24 h of admission), the resuscitation ratios of both FFP/RBC and PLT/RBC are undoubtedly important in reducing hemorrhagic mortality. However, neither civilian nor military practice has yielded a consensus on optimal PLT transfusions.

**Fig. 2:**
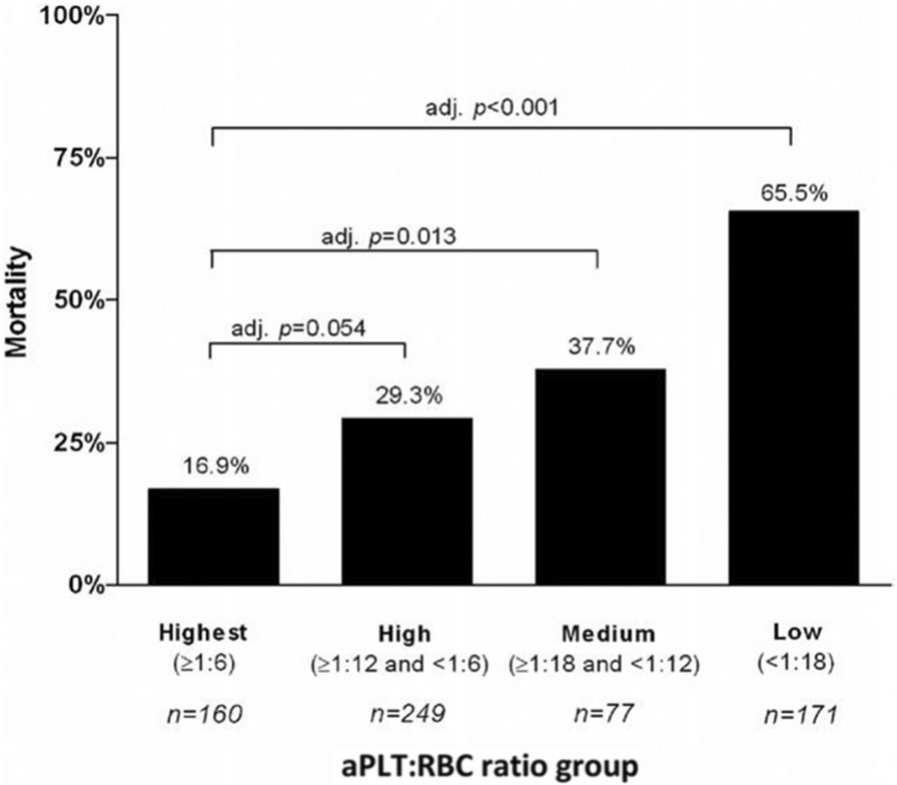
Mortality of massively transfused patients at 24 h stratified by platelet ratio. Adjusted for hypotension on admission (90 vs. 90 mmHg), GCS on admission (8 vs. 8), FFP/RBC ratio (%) at 24 h, and cryoprecipitate at 24 h. *FFP* fresh frozen plasma, *GCS* Glasgow Coma Scale, *RBC* red blood cell

**Fig. 3:**
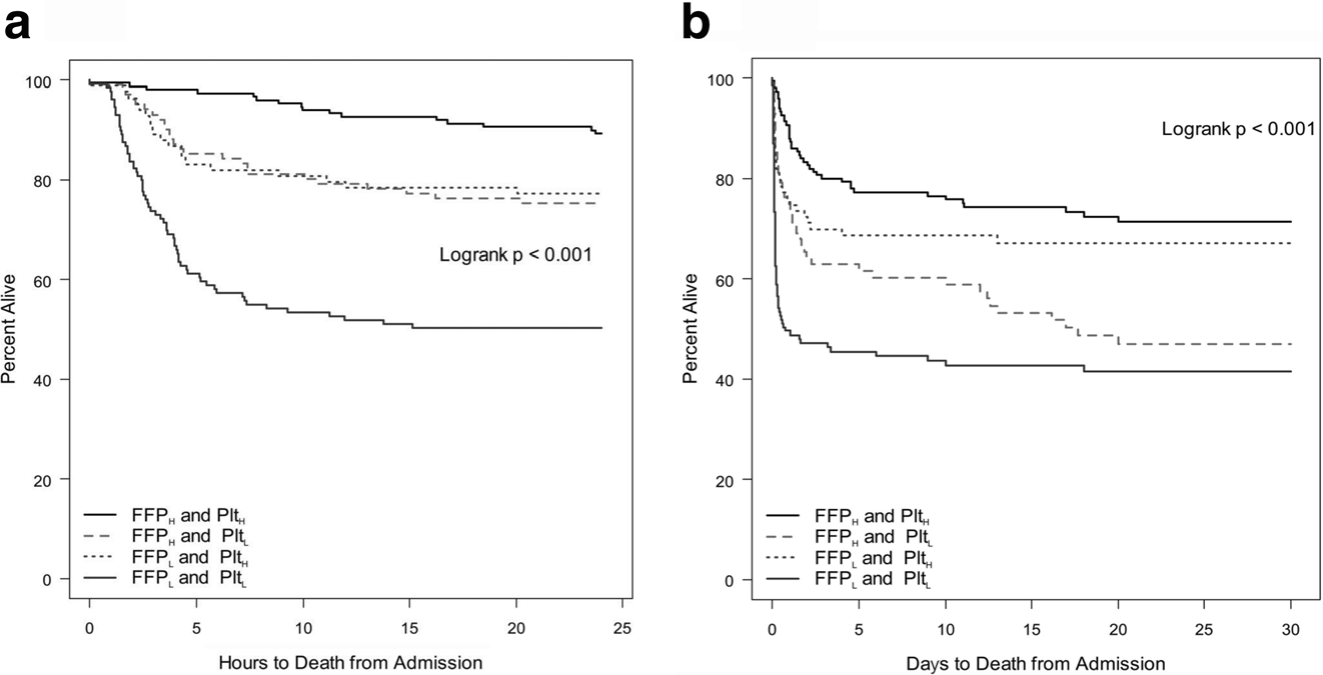
Kaplan–Meier survival plot for the first **a** 24 h and **b** 30 days after admission. **a** Kaplan–Meier survival plot for the first 24 h after admission for the four groups (high plasma (*FFP*_*H*_) or platelet (*Plt*_*H*_) to RBC ratio 1:2, low plasma (*FFP*_*L*_) or platelet (*Plt*_*L*_) to RBC ratio 1:2). **b** Kaplan–Meier survival plot for the first 30 days after admission for the four groups (high plasma (*FFP*_*H*_) or platelet (*Plt*_*H*_) to RBC ratio 1:2, low plasma (*FFP*_*L*_) or platelet (*Plt*_*L*_) to RBC ratio 1:2). *FFP* fresh frozen plasma, *RBCs* red blood cells

**Fig. 4:**
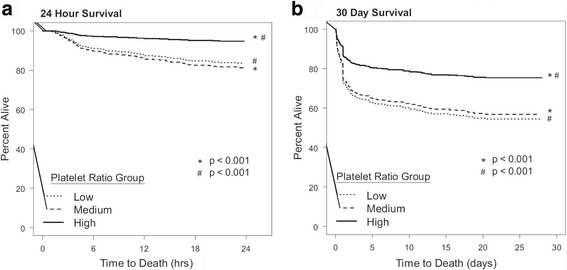
Propensity-adjusted Kaplan–Meier survival plot for the first **a** 24 h and **b** 30 days after admission. **a** Propensity-adjusted Kaplan–Meier survival plot for the first 24 h after admission for the three platelet ratio groups: low (1:20), medium (1:2), and high (1:1). **b** Propensity-adjusted Kaplan–Meier survival plot for the first 30 days after admission for the three platelet ratio groups: low (1:20), medium (1:2), and high (1:1)

Inaba et al. [38] evaluated the impact of PLT transfusion in trauma patients receiving massive transfusion. With a decreasing PLT/RBC ratio, mortality at 24 h increased in a stepwise fashion. Compared with the group with the highest ratio (>1:6), the adjusted relative risk of death was 1.67 (adjusted *p* = 0.054) in the high-ratio group (≥1:12 and <1:6), 2.28 (adjusted *p* = 0.013) in the medium-ratio group (≥1:18 and <1:12), and 5.51 (adjusted *p* < 0.001) in the low-ratio group (<1:18). A similar stepwise increase in mortality with a decreasing PLT/RBC ratio was observed at 12 h after admission. After stepwise logistic regression, a high PLT/RBC ratio was independently associated with improved survival at 24 h (adjusted *p* < 0.001) (Fig. 2). Holcomb et al. [10] also determined the effect of the blood component ratio in massive transfusion. Patients with a high PLT/RBC ratio (≥1:2) had a significantly higher 30-day survival than those with a low PLT/RBC ratio (<1:2) (high 59.9% vs. low 40.1%, *p* < 0.01) as did those with a high (≥1:2) vs. low (<1:2) FFP/RBC ratio (high 59.6% vs. low 40.4%, *p* < 0.01). The authors concluded that not only a PLT/RBC ratio of ≥1:2 but also an FFP/RBC ratio of ≥1:2 was shown to be optimal and that both ratios were independent predictors of death at 6 and 24 h and at 30 days. These two treatment groups were then expanded to four in a 2 × 2 factorial layout (group 1: high FFP and PLT ratio; group 2: high FFP and low PLT ratio; group 3: low FFP and high PLT ratio; and group 4; low FFP and low PLT ratio). Kaplan–Meier analysis showed a significant separation of the groups within 6 and 24 h (*p* < 0.001) and that survival was higher in group 1 than in the other groups, at both 6 and 24 h (*p* < 0.001) (Fig. 3a). The overall 24-h difference was sustained for 30 days (*p* < 0.001) (Fig. 3b). These results suggest that in severe trauma patients the survival rate depends on a high PLT/RBC ratio rather than a high FFP/RBC ratio. Additionally, Holcomb et al. [39] retrospectively investigated the relationship between the PLT/RBC ratio and outcome based on 643 trauma patients who received massive transfusion. The patients were divided into three groups: a low-ratio group (>1:20), a medium-ratio group (1:2), and a high-ratio group (1:1). A propensity-adjusted Kaplan–Meier survival plot showed that higher PLT ratios were associated with improved survival at 24 h and 30 days (*p* < 0.001 for both) (Fig. 4).

Johansson et al. [2] performed a meta-analysis of the potential effect on survival of hemostatic resuscitation based on the proactive administration of PLTs rather than RBCs in trauma patients with massive bleeding. Two studies addressed the effect of high vs. low PLT transfusion rates in 641 massively bleeding trauma patients, of whom 333 received high PLT/RBC ratio [40, 41]. According to these two studies, patients receiving PLT/RBC in a high ratio had a significantly lower mortality (odds ratio 0.45, 95% confidence interval 0.37–0.55). This finding could not be attributed to the heterogeneity between the studies (*I* ^2^ = 0%). However, because no randomized studies evaluating the effect of different transfusion ratios were included in this report, the evidence level of this meta-analysis was low. Additionally, the absence of a relationship between the PLT/RBC ratio and overall mortality has been reported [42, 43].

After considering the above findings, the administration of at least one pool of PLTs (four to six individual donor units) for every five units of RBCs to trauma patients requiring massive transfusion seems reasonable [44].

## Combined therapy with RBC, FFP, and PLT

Previous investigations of the FFP/RBC transfusion ratio supported a ratio of 1:1 or higher [10, 45]. However, in the management of ATC/TIC, the simultaneous administration of not only RBCs and FFP but also PLTs, in appropriate ratios, is recommended, with limited use of crystalloid or colloid solution.

The “optimal” ratio is the subject of ongoing debate because it may be complicated by the volumes of anticoagulant and RBC additive solution in modern blood components. In the abovementioned study by Kornblith et al. [23], the differences in the INR, PTT, and PLT counts obtained with whole blood vs. reconstituted blood composed of either 1:1:1 or 2:1:1 ratios of units of RBC, FFP, and PLT were investigated (Fig. 1). The results of the present study showed that the mean INR of 1:1:1 reconstituted blood was 1.31 and the mean PTT 42 s (1.4 times > normal), whereas the values for 2:1:1 reconstituted blood were 1.55 and 46 s (1.53 times > normal), respectively. Moreover, PLT counts were higher for 1:1:1 than for 2:1:1 blood components (129 × 10^9^/L vs. 95 × 10^9^/L, respectively), and typically, only 70% of the transfused PLTs circulated (Table 1). This simple physical consequence of mixing blood products suggests that treatment with 1:1:1 blood components has the greater potential to correct ATC/TIC [26].

## The potential of FFP/PLT/RBC = 1:1:1

In the last decade, an alternative resuscitation strategy was developed based on providing only the conventional blood components FFP, PLT, and RBC in a 1:1:1 ratio to maintain the intravascular volume, oxygen-carrying capacity, plasma coagulation factors, and functioning platelets. The administration of crystalloid fluids was markedly limited, and other colloid-containing fluids given for massive bleeding were avoided. In severely injured patients, this strategy appears to not only save lives but also to reduce blood product consumption [26]. In light of this result, many guidelines now recommend 1:1:1 ratios.

Nascimento et al. [46] reported a feasibility study based on a small randomized controlled trial that included trauma patients expected to require a massive transfusion. A fixed FFP/PLT/RBC ratio of 1:1:1 was compared with standard practice (laboratory result-guided transfusion protocol). The trial was able to achieve the 1:1:1 ratio in 57% (21 of 37) of the patients in the fixed ratio group compared with 6% (2 of 32) of those in the control group, thus demonstrating the feasibility of the intervention. While the study was not powered to detect a difference in mortality, the all-cause 28-day mortality by intention-to-treat analysis (relative risk for fixed ratio, 2.27; 95% confidence interval, 0.98–9.63) and by per-protocol analysis (relative risk for fixed ratio, 3.17; 95% confidence interval, 1.15–18.24) was consistent with a safe outcome.

The recent Pragmatic Randomized Optimal Plasma and Platelet Ratios (PROPPR) trial [47], a large, prospective, randomized, interventional trial in which patients with severe bleeding trauma were the focus compared the efficacy and safety of a 1:1:1 transfusion ratio of FFP/PLT/RBC to a 1:1:2 ratio. There was no difference in 24-h or 30-day mortality between the two groups. However, a 1:1:1 ratio did result in a significant reduction in mortality from bleeding within the first 24 h (9.2 vs. 14.6%; *p* = 0.03), with no increase for the 1:1:2 ratio group in ARDS, venous thromboembolism, or other transfusion-related complications. The absolute benefit was a 4% reduction in mortality among randomized patients, and the relative benefit a 15% reduction in total mortality, from 26 to 22% overall.

However, the prospective observational PROMMTT trial [3] emphasized anew the problems arising from bias in the blood product delivery time. Despite the fact that all 10 participating centers tried to deliver the products in a 1:1:1 ratio, the fraction that succeeded in achieving this ratio for plasma was 30% at 1 h, 40% at 2 h, and 50% at 6 h. The achieved ratios were even worse for PLTs [17].

## Conclusion

Preventable trauma death may be attributable to the absence of appropriate initial resuscitation for bleeding. Therefore, any effective resuscitation strategy must be designed to complement the appropriate and prompt correction of anemia, coagulopathy, and abnormalities in fibrinolysis. In DCR, transfusion must be carried out during the early stage of patient management. It involves the use of increased amounts of plasma and PLTs along with the first units of RBCs, while simultaneously minimizing crystalloid administration (1000-2000 mL) in patients who are predicted to require massive transfusion. At the present time DCR is recommended for rapid hemorrhage control through early administration of a mixture of FFP, PLTs, and RBCs in a balanced ratio of 1:1:1.

## Abbreviations

aPTT: Activated partial thromboplastin time
ATC: Acute traumatic coagulopathy
DCR: Damage control resuscitation
FFP: Fresh frozen plasma
PLT: Platelet
PT: Prothrombin time
PTD: Preventable trauma death
RBC: Red blood cell
TIC: Trauma-induced coagulopathy
